# Temperature effect on polymerase fidelity

**DOI:** 10.1016/j.jbc.2021.101270

**Published:** 2021-10-23

**Authors:** Yuan Xue, Ido Braslavsky, Stephen R. Quake

**Affiliations:** 1 Department of Bioengineering, Stanford University, Stanford, California, USA; 2 The Robert H. Smith Faculty of Agriculture, Food and Environment, Institute of Biochemistry, Food Science and Nutrition, The Hebrew University of Jerusalem, Rehovot, Israel; 3 Department of Applied Physics, Stanford University, Stanford, California, USA; 4 Chan Zuckerberg Biohub, Mission Bay, California, USA

**Keywords:** polymerase fidelity, psychrophilic DNA polymerase, temperature effect on enzymes, polymerase error rate, KleExo-, KleLF lacking the 3′→5′ exonuclease domain, KleLF, Klenow fragment, MBP, maltose-binding protein, PIPI, *Psychromonasingrahamii*DNA polymerase I, UMIs, unique molecular identifiers

## Abstract

The discovery of extremophiles helped enable the development of groundbreaking technology such as PCR. Temperature variation is often an essential step of these technology platforms, but the effect of temperature on the error rate of polymerases from different origins is underexplored. Here, we applied high-throughput sequencing to profile the error rates of DNA polymerases from psychrophilic, mesophilic, and thermophilic origins with single-molecule resolution. We found that the reaction temperature substantially increases substitution and deletion error rates of psychrophilic and mesophilic DNA polymerases. Our motif analysis shows that the substitution error profiles cluster according to phylogenetic similarity of polymerases, not the reaction temperature, thus suggesting that the reaction temperature increases the global error rate of polymerases independent of the sequence context. Intriguingly, we also found that the DNA polymerase I of psychrophilic bacteria exhibits higher polymerization activity than its mesophilic ortholog across all temperature ranges, including down to −19 ^°^C, which is well below the freezing temperature of water. Our results provide a useful reference for how the reaction temperature, a crucial parameter of biochemistry, can affect DNA polymerase fidelity in organisms adapted to a wide range of thermal environments.

Micro-organisms that live in cold environment are confronted with the thermodynamic challenge of maintaining the chemical processes of life; however, the abundance of various life-forms found in near or below freezing temperatures suggests that biology has evolved ways to overcome such obstacles. Contrary to what one would expect from naïve application of the Arrhenius equation, micro-organisms in cold environments do not grow exponentially slower in the cold than their mesophilic cousins at room temperature (1, 2). This suggests that psychrophilic enzymes have evolved to catalyze relevant activities at low temperature (3, 4, 5). Low-temperature adaptation is thought to have increased the catalytic rate of enzymes by increasing local flexibility of their active sites, thus lowering activation energy barriers (6, 7, 8, 9). This can be contrasted with high-temperature adaptation, which confers structural robustness to thermal denaturation by increasing intramolecular interactions that maintain an enzyme's functional tertiary shape. Studies based on directed evolution have shown that structural stability and enzymatic efficiency can be optimized simultaneously and are thought to be mutually compatible properties (10, 11). Combining these observations helps explain the general biochemical trend of cold-adapted enzymes: an increase in enzyme’s catalytic activity is often associated with a reduced binding affinity and a broader substrate specificity (12, 13). However, a direct test of these ideas on orthologous enzymes from organisms adapted to low, medium, and high temperatures has yet been performed.

As DNA polymerase is a central component of cellular replication and biotechnological platforms (14), understanding how the reaction temperature can affect fidelity is vital. The polymerase error rate is tightly controlled to ensure the successful duplication of genetic material. Replication error from DNA polymerase is a source of genetic variations and underlies the cause of many diseases (15, 16, 17, 18). Polymerase errors also play a crucial role in biotechnology applications such as PCR and multiple-displacement amplification (19, 20, 21). Polymerase can introduce errors at an exponential rate, significantly impairing downstream interpretation. In high-throughput sequencing, polymerase replication errors can reduce base calling accuracy and precision (22, 23, 24). The increasing applications of nucleic acid amplification underscore a need to better understand the degree to which physical parameters, such as the temperature, affects polymerase fidelity.

This study set out to provide a reference for how the temperature affects the activity and error profiles of DNA polymerases of psychrophilic, mesophilic, and thermophilic origins (Fig. 1*A*). Previous studies reported psychrophilic DNA polymerases' activity at ambient temperatures (25, 26); however, the behavior of these polymerases across a wide range of reaction temperatures remains unexplored. In this study, we demonstrate for the first time that DNA polymerase I from psychrophilic bacteria, *Psychromonas ingrahamii*, retains DNA replication activity below water's freezing temperature. We used random nucleotides to multiplex single-molecule measurement of insertion, deletion, and substitution events for DNA polymerases from different origins across a range of temperature conditions. This enabled a comprehensive mapping of DNA polymerase error profiles as a function of the reaction temperature, revealing an unexpected relationship between fidelity and temperature adaptation. Our report provides an important reference to inform design choices for future biotechnological applications of DNA polymerase.

**Figure 1:**
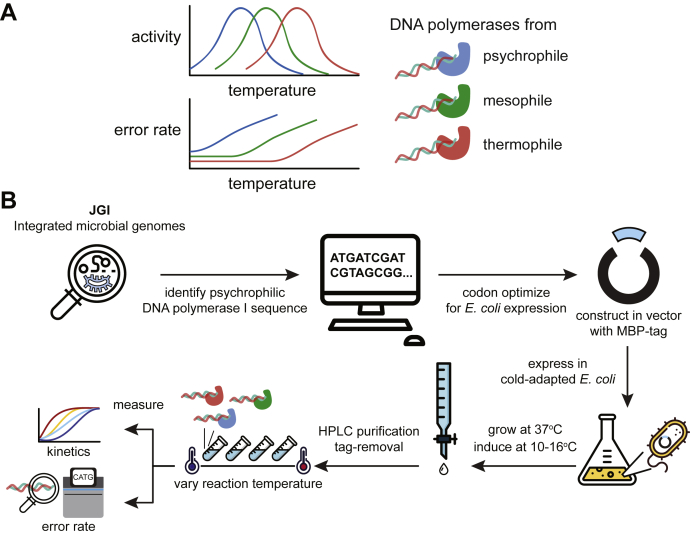
**Schematic overview of this study.***A*, graphical summary of the study. *B*, a schematic illustrating the apparent trade-off between enzymatic speed and fidelity in the context of temperature adaptation. Thermophilic enzymes are expected to be selected for a low error rate due to increased environmental temperature. Psychrophilic enzymes are expected to be selected for a high enzymatic speed due to decreased environmental temperature.

## Results

### Psychrophilic DNA polymerase is more active than mesophilic and thermophilic polymerases and can replicate DNA below zero degrees Celsius

To address whether there is an activity and fidelity trade-off in DNA polymerase, we wanted to measure and compare the temperature dependence of its activity from a wide range of microbial sources. As psychrophilic DNA polymerase is unavailable commercially, we set out to recombinantly purify one for our study. Based on homology search, we identified a gene encoding DNA polymerase I (*Psychromonas* *ingrahamii* DNA polymerase I [PIPI]; IMG Gene ID: 639798289), a family A polymerase, from *P. ingrahamii,* gram-negative bacteria that can grow and replicate at −12 ^°^C in laboratory condition (27, 28, 29). Maltose-binding protein (MBP)-PIPI was purified by maltose affinity column, cleaved off its tandem-fused MBP tag, and then repurified with size-exclusion chromatography (Experimental procedures) (Fig. S1*A*). A graphical overview of our study is summarized in Figure 1*B*.

**Figure 2:**
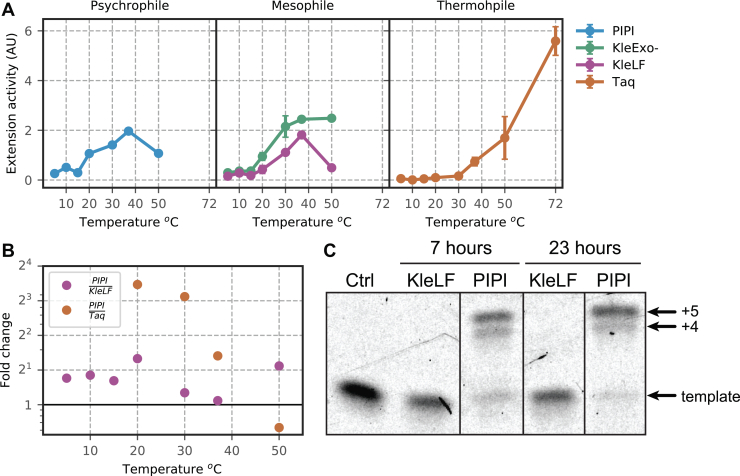
**Extension activity of DNA polymerases across a wide range of temperature.***A*, extension activity of purified psychrophilic, mesophilic, and thermophilic DNA polymerases. *B*, fold change of PIPI extension activity relative to KleLF or Taq. Taq activity is indistinguishable from background below 20 ^°^C, so comparison to its activity is excluded at a low temperature range. *C*, PIPI and KleLF are prediluted to an equal concentration, and they are coincubated with a primed template at −19 ^°^C for 7 or 23 h. The DNA template (22 bp) is hybridized with a complementary 17-bp-long 5′-ATTO 633–labeled oligonucleotide, leaving a 5-bp gap at the 3′ end. KleLF, Klenow fragment; PIPI, *Psychromonas* *ingrahamii* DNA polymerase I.

To determine the temperature profile of polymerase extension activity, we measured the extension rate of DNA polymerase I of psychrophilic, mesophilic, and thermophilic origins across a range of incubation temperatures. Briefly, we use a fluorometric assay to monitor the level of fluorescence over time at different incubation temperatures. When the polymerase extends from the primed template, we can detect an increase of fluorescence emission due to binding of dsDNA to the EvaGreen fluorophore. As the fluorescence level is linearly proportional to the amount of dsDNA product, we can thus infer the polymerase extension activity by calculating the initial rate of fluorescence change per unit time (Experimental procedures). We were able to detect robust activity in the recombinantly purified PIPI (left panel in Fig. 2*A*), suggesting it is biochemically active. Similar to its mesophilic ortholog, the Klenow fragment (KleLF), PIPI exhibits peak activity at around 37 ^°^C. This is consistent with previous reports that the optimal temperature of enzyme activity can differ from the temperature to which the host organism is adapted (26, 30). The KleLF lacking the 3′→5′ exonuclease domain (KleExo-) exhibits higher activity across all temperature ranges, suggesting that the observed activity may also be dependent on the presence of the exonuclease domain. As expected, Taq DNA polymerase is inactive at low temperatures below 30 ^°^C and its activity continues to increase at up to 72 ^°^C. Strikingly, PIPI exhibits higher extension activity than KleLF at or below 37 ^°^C (Fig. 2*B*). The fold difference in activity between PIPI and Taq is even more dramatic, displaying over a 10-fold difference at 30 ^°^C. PIPI activity decreases with an increasing reaction temperature because of thermal inhibition. Our observation is thus consistent with the hypothesis that enzymes of psychrophilic origin are evolved to have higher activity, even at moderate temperatures.

Previous reports showed that *P. ingrahamii* could grow and replicate at negative 12 ^°^C (28). We thus reasoned that PIPI should remain active below 0 ^°^C. Using 5′-fluorescently labeled primers (Experimental procedures), we performed a gel-based primer extension assay to measure the activity of PIPI and the KleLF at −19 ^°^C in an aqueous solution that had been supplemented with 30% glycerol. Strikingly, we discover that PIPI retains extension activity as it completed extension of a primed template with a 5-nucleotide gap under 7 h, whereas the KleLF did not (Fig. 2*C*). The KleLF failed to complete extension at −19 ^°^C up to 23 h (Fig. 2*C*). We wondered whether this is due to inhibition of KleLF binding to dsDNA. By modeling the thermodynamics of binding in previous data, we found that the KleLF binds to dsDNA favorably at down to −19 ^°^C (ΔG: −9.48 kcal/mol) (31). This suggests the KleLF is catalytically inactivated at low temperatures, as suggested by other studies (30, 32). In the later part of this article, we discuss several exciting possibilities of utilizing PIPI for subzero reactions that may open the door for novel biochemical applications.

### Reaction temperature increases the error rate of DNA polymerases

**Figure 3:**
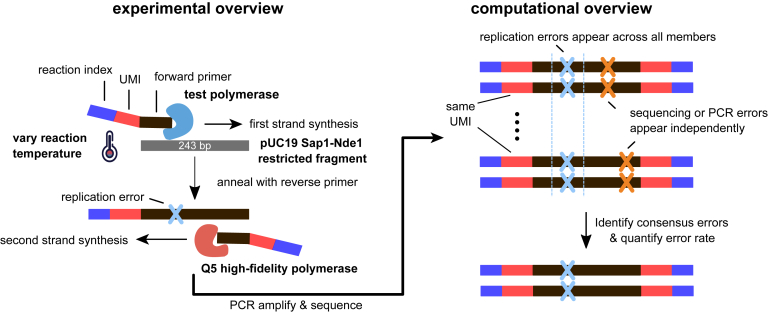
**Schematic overview of single-molecule polymerase error measurement.***A*, a schematic illustrating the experimental overview of multiplexed single-molecule measurement of the polymerase error rate at various reaction temperatures. Replication errors (highlighted by *blue crosses*) are distinguished from sequencing errors (highlighted by *orange crosses*) by comparing the consensus sequence of individual molecules with the reference sequence in the pUC19 Sap1-Nde1 fragment.

To profile polymerase errors across a range of reaction temperatures and conditions, we adapted a high-throughput multiplexed sequencing approach (33). Briefly, a reaction mixture that contains a complementary primer and ssDNA pUC19 fragment (NEB) is equilibrated to the desired temperature before the addition of polymerase. Unless otherwise noted, the mixture is buffered against 3-(*N*-morpholino)propanesulfonic acid (Mops, pH 8.5) whose pKa has relatively small temperature dependence. It is essential to control for changes in pH as this can influence enzyme fidelity independently of the temperature (34). After quenching, dsDNA products are PCR-amplified, purified, and pooled at equimolar concentrations for sequencing on a MiSeq 2 × 300 bp platform (Experimental procedures). We found no runoff amplification by-products in up to 26 rounds of PCR amplification, while 20 rounds of PCR amplification failed to yield sufficient DNA amount for downstream purification (Fig. S2*A*). The purified libraries are pooled and sequenced on the Illumina MiSeq platform. We deconvolved the sequenced reads by their reaction indices and unique molecular identifiers (UMIs), removed sequencing errors by trimming reads with low quality, and called consensus sequence of molecules. Quantification of base counts with high sequence consensus reveals uniform coverage along the entire pUC19 template, indicating no substantial positional effect on measurement dropout. An overview of the experimental and analytical workflows is illustrated in Figure 3*A*.

**Figure 4:**
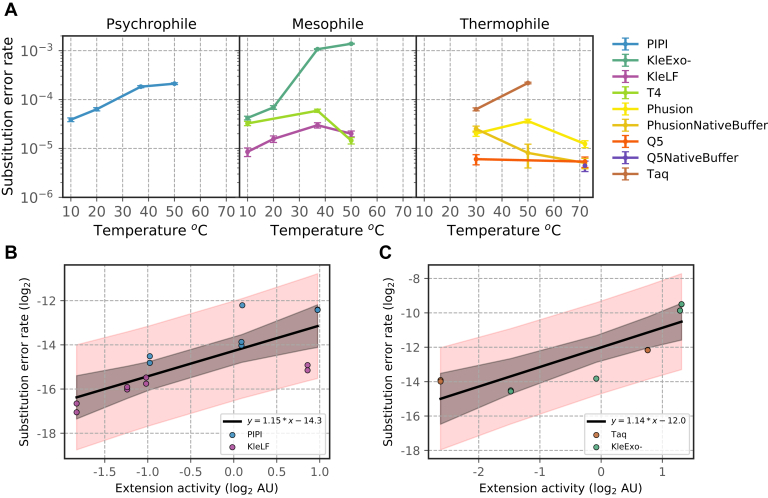
**Analysis of DNA polymerase activity-fidelity tradeoff.***A*, substitution error rates (per base) of psychrophilic (*left*), mesophilic (*center*), and thermophilic (*right*) DNA polymerases as a function of the reaction temperature. Error bars are the SD of the mean error rate fitted to a binomial distribution. Linear regression of substitution error rate on extension activity for polymerases with predicted exonuclease activity (*B*) (PIPI and KleLF) and (*C*) for ones without (Taq and KleExo-). *Shaded* regions indicate 95% (*black*) prediction intervals of linear regression and (*red*) confidence intervals of the sampling mean. KleExo-, KleLF lacking the 3′→5′ exonuclease domain; KleLF, Klenow fragment.

We categorized replication error events as substitution, deletion, or insertion error and quantified the frequency of these error types in each unique molecule. As insertion and deletion events are relatively rare, we focused our analysis primarily on substitution error of psychrophilic, mesophilic, and thermophilic DNA polymerases in Figure 4*A*. A comprehensive report of error rates and coverage for thermophilic, psychrophilic, and mesophilic DNA polymerases is presented in Table 1, Table 2, Table 3. Quantification of the Q5 DNA polymerase error rate in its native buffer (Q5NativeBuffer) at 72 ^°^C provides a baseline for our measurement, revealing an average substitution, deletion, and insertion rates of 4.44 × 10^−6^, 9.50 × 10^−7^, and 1.37 × 10^−7^ per base, respectively, which agrees well with previously reported values (Table 1) (33, 35). Phusion (Thermo Fisher), another high-fidelity and commonly used thermophilic polymerase, has a similar substitution rate of 5.05 × 10^−6^ per base (Table 1) at 72 ^°^C in its native Phusion HF buffer (PhusionNativeBuffer; Thermo Fisher). We discover that the substitution rate of Phusion DNA polymerase increases by more than a factor of two when switched from its native buffer to Mops; furthermore, we notice an increased substitution rate of Phusion polymerase in its native buffer (PhusionNativeBuffer) in lower reaction temperatures. We suspect the native buffer of Phusion polymerase (Thermo Fisher) is designed for a specific temperature and its pH is highly temperature dependent. In low temperatures, the pH changes in the buffer system cause suboptimal polymerase fidelity. This would explain why the substitution rate of Phusion in the Mops buffer remains relatively invariant to temperature changes (Table 1 and right panel in Fig. 4*A*). Supporting this hypothesis, we do not observe substantial differences in the substitution rate of Q5 polymerase in the Mops buffer between 30 ^°^C and 72 ^°^C. Our finding strongly suggests that thermophilic polymerases from commercial sources should use alternative buffers to ensure optimal fidelity at low temperatures.

**Table 1:** Error rates (per base) for thermophilic DNA polymerases

Origin	Reaction	Temperature (°C)	Substitution rate (base^−1^)	Deletion rate (base^−1^)	Insertion rate (base^−1^)	Bases counted
Thermophile	Q5^NB^	72	4.44 × 10^−6^	9.50 × 10^−7^	1.37 × 10^−7^	15,536,120
	Q5	30	6.04 × 10^−6^	7.45 × 10^−7^	5.31 × 10^−7^	6,001,934
	Q5	72	5.38 × 10^−6^	ND	ND	6,340,445
	Taq	30	6.32 × 10^−5^	2.27 × 10^−6^	2.01 × 10^−6^	6,497,391
	Taq	50	2.18 × 10^−4^	2.18 × 10^−5^	3.53 × 10^−6^	6,320,921
	Phusion^NB^	30	2.51 × 10^−5^	ND	8.32 × 10^−7^	2,064,657
	Phusion^NB^	50	8.11 × 10^−6^	ND	ND	478,302
	Phusion^NB^	72	5.05 × 10^−6^	ND	ND	10,515,198
	Phusion	30	2.02 × 10^−5^	2.32 × 10^−6^	4.81 × 10^−7^	6,479,246
	Phusion	50	3.64 × 10^−5^	2.46 × 10^−6^	4.84 × 10^−7^	7,260,714
	Phusion	72	1.24 × 10^−5^	ND	5.11 × 10^−7^	6,182,116

NB denotes the reaction carried out in the native buffer supplied by the manufacturer.

ND denotes the error frequency below the detectable error baseline.

**Table 2:** Error rates (per base) for psychrophilic DNA polymerase

Origin	Reaction	Temperature (°C)	Substitution rate (base^−1^)	Deletion rate (base^−1^)	Insertion rate (base^−1^)	Bases counted
Psychrophile	PIPI	10	3.87 × 10^−5^	1.48 × 10^−6^	1.62 × 10^−7^	6,517,110
	PIPI	20	6.34 × 10^−5^	4.15 × 10^−6^	2.20 × 10^−7^	5,630,032
	PIPI	37	1.83 × 10^−4^	1.59 × 10^−5^	ND	4,747,033
	PIPI	50	2.11 × 10^−4^	8.47 × 10^−6^	ND	2,759,232

ND denotes the error frequency below the detectable error baseline.

**Table 3:** Error rates (per base) for mesophilic DNA polymerases

Origin	Reaction	Temperature (°C)	Substitution rate (base^−1^)	Deletion rate (base^−1^)	Insertion rate (base^−1^)	Bases counted
Mesophile	KleExo-	10	4.20 × 10^−5^	9.50 × 10^−7^	ND	6,370,293
	KleExo-	20	6.91 × 10^−5^	7.45 × 10^−7^	5.31 × 10^−7^	2,000,239
	KleExo-	37	1.07 × 10^−3^	ND	ND	3,472,537
	KleExo-	50	1.38 × 10^−3^	2.27 × 10^−6^	2.01 × 10^−6^	3,408,561
	KleLF	10	8.53 × 10^−6^	2.18 × 10^−5^	3.53 × 10^−6^	5,822,486
	KleLF	20	1.56 × 10^−5^	ND	8.32 × 10^−7^	5,522,080
	KleLF	37	2.99 × 10^−5^	ND	ND	4,317,523
	KleLF	50	2.00 × 10^−5^	ND	ND	5,415,816
	T4	10	3.25 × 10^−5^	2.32 × 10^−6^	4.81 × 10^−7^	6,168,596
	T4	37	5.92 × 10^−5^	2.46 × 10^−6^	4.84 × 10^−7^	6,093,149
	T4	50	1.45 × 10^−5^	ND	5.11 × 10^−7^	6,117,463

ND denotes the error frequency below the detectable error baseline.

Intriguingly, we observe a substantial temperature dependence for psychrophilic and mesophilic polymerase substitution error rates. The effect is most dramatic on PIPI and KleExo- whose substitution rate rose by more than 5- and 30-fold, respectively, between reaction temperatures of 10 ^°^C and 50 ^°^C (Tables 2 and 3) (left and center panels in Fig. 4*A*). Similarly, the deletion rate also scales positively with the reaction temperature for PIPI and KleExo- (Tables 2 and 3) (Fig. S2*B*). Curiously, the temperature effect on the substitution error rate of the KleLF and T4 polymerases is not monotonic, revealing a maximum substitution rate at 37 ^°^C (Table 3) (center panel in Fig. 4*A*). This is consistent with the observation that T4 DNA polymerase's error rate can decrease at a higher temperature (36). Comparison of KleLF and KleExo- suggests that the presence of an exonuclease domain may have a dominant and inhibitory effect on the error rate at a high reaction temperature.

To investigate whether there is an activity-fidelity trade-off, we quantified the relationship between the extension activity and substitution error rate for polymerases with and without exonuclease activity using linear regression model (Fig. 4, *B* and *C*). For PIPI and KleLF, which are predicted to exhibit exonuclease activity, we found that extension activity (log_2_) significantly predicted the substitution error rate (log_2_), *b* = 1.1502, *t*(14) = 4.385, *p* < 0.001. Strikingly, we observed a similar relationship in Taq and KleExo- polymerases that lack exonuclease activity, *b* = 1.1394, *t*(10) = 5.088, *p* < 0.01. Intuitively, our model predicts that for every doubling of polymerase activity, the polymerase error rate increases by about a factor of 2.2. Extension activity (log_2_) also explained a significant portion of variance in the substitution error rate (log_2_), *R*^2^ = 0.597, *F*(1, 13) = 19.23, *p* < 0.001 (Fig. 4*B*), and *R*^2^ = 0.742, *F*(1, 9) = 25.89, *p* < 0.001 (Fig. 4*C*). We next analyzed the changes in substitution error spectrum across the reaction temperatures. The reaction temperature had the most substantial effect on the substitution spectrum of KleExo-. As expected, an increase in the reaction temperature is associated with an increased frequency of transversion relative to transition errors (Fig. S2*C*). Meanwhile, the reaction temperature had a relatively mild effect on the substitution spectrum of other polymerases that we investigated.

### Distinct mutational profiles are driven by the polymerase family type

**Figure 5:**
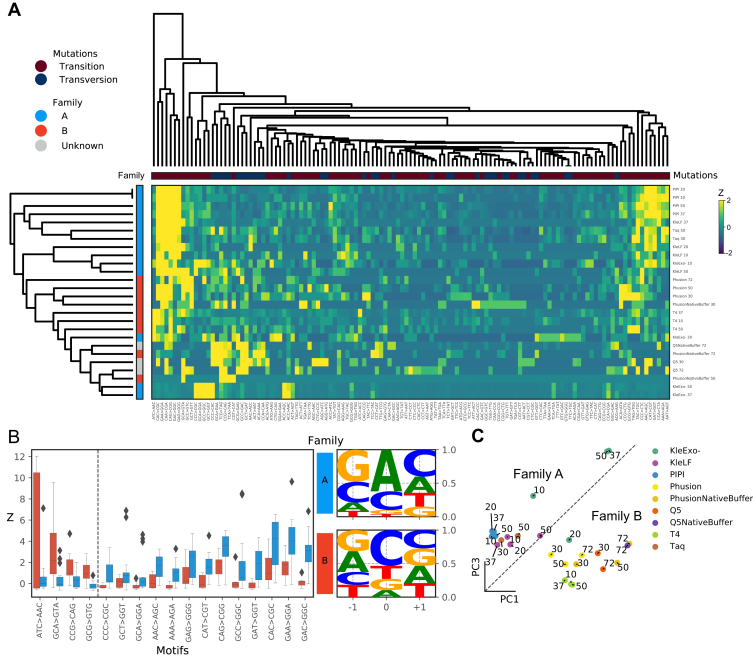
**DNA polymerase substitution error motif analysis.***A*, hierarchical clustering of the standardized 3-mer substitution rates naturally reveals clustering of the error motif by the phylogenetic origins of the polymerase. *B*, substitution motifs that most distinguish errors made by family A and family B polymerases. Logo plots quantify the frequency of substitution motifs at −1, 0, and +1 positions that are generated by polymerase families A (*top*) or B (*bottom*). *C*, projection of polymerase reactions by principal components (PCs) 1 and 3 of standardized 3-mer substitution rates.

The polymerase replication error rate is context dependent (37, 38). We wondered whether phylogenetically related polymerases that experienced vastly different temperature adaptation would produce substitution profiles similar to each other. We also asked whether these profiles varied across reaction temperatures and polymerase phylogeny. To validate the phylogenetic relations, we aligned the primary peptide sequences of PIPI to polymerases with known family assignment (Fig. S3*A*). To analyze the relations between polymerase family and sequence context of substitution errors, we generated a 3-mer substitution spectrum for each condition. The entire spectrum consists of 192 permutations (4 possible nucleotides each at −1, 0, +1 positions, and three possible mutated bases). To enrich for informative motif, we kept 3-mer substitutions that occurred at greater than 20% frequency in at least a single reaction and removed the rest from further analysis. This filtering step allows us to narrow down to 60 distinct 3-mer substitutions (∼31.3% of the entire 3-mer spectrum) and focus on substitutions that are common in at least some of the reactions. The hierarchical clustering of the filtered 3-mer substitution matrix reveals substitution profiles generated by the same polymerase cluster across different reaction temperatures. This indicates that the effect of the reaction temperature on polymerase substitution error is independent of the sequence context (Fig. 5*A*). Interestingly, we discover that the substitution profiles tended to cluster by the phylogenetic distance of the polymerases in which substitutions generated by families A and B largely separated into distinct clusters regardless of their respective temperature adaptation range.

To identify mutational profiles that best explain the observed substitution profiles, we trained a random forest decision tree to classify the polymerase family origin of each profile. By ranking the learned weight for each feature, we identified the most distinguishing mutational footprints for each polymerase family (Fig. 5*B*). We discover that polymerase family A more frequently produced A → G transition when preceded by G/C at the −1 position. On the other hand, polymerase family B more frequently produced pyrimidine (C/T) → a transversion when preceded by a purine (A/G) at the −1 position. We then performed principal component analysis on the standardized 3-mer error rate. This led us to identify principal components 1 and 3 that best explained the polymerase families' substitution profiles. Overall, a high principal component 1 score is associated with the substitution profile of family B, while high principal component 3 score is associated with that of family A. Projection of the substitution profiles onto principal components 1 and 3 reveals a natural decision boundary on this space that separates the substitution error spectrum between family A and B (Fig. 5*C*). This suggests that conserved structural differences between the two polymerase families may underlie polymerase's propensity to generate distinct mutational signatures independent of temperature adaptation experience and the reaction temperature.

## Discussion

DNA polymerases are the biological machinery underlying the transmission of genetic information and hold the key to many biotechnological innovations. While most studies have focused on polymerases of mesophilic or thermophilic origins, this is one of the first to provide a comprehensive biochemical characterization of activity and fidelity of bacterial DNA polymerases across a wide temperature-adaptation range. We characterized the temperature dependence of apparent replication activity in DNA polymerases. Earlier studies had reported that mesophilic and thermophilic DNA polymerases become increasingly inactivated at a low temperature range (30, 32). Consistent with their reports, we were unable to detect DNA replication activity of the KleLF at negative 19 ^°^C. Intriguingly, we detected robust activity with the psychrophilic DNA polymerase PIPI. To our knowledge, this is the first demonstration that DNA extension can be accomplished with a psychrophilic DNA polymerase below water's freezing temperature (0 ^°^C). As these two polymerases are phylogenetic cousins, comparative study into the molecular mechanism for why the psychrophilic polymerase retains activity at a low temperature may yield additional insights into the design principles of enzymes. A low temperature reaction is desirable as it can inhibit nuclease activity and reduce phototoxicity and DNA damages (24, 39). Reactions in exotic environments such as the arctic and outer space may also require polymerases that can function at the low temperature range. The ability to engineer psychrophilic polymerases can thus enable nucleic acid amplification techniques directly on cryopreserved samples or in cold environment, reducing the risk of contamination and signal degradation (40). Combining polymerases adapted to different temperature ranges can, in principle, enable thermally multiplexed nucleic acid reaction that can substantially reduce assay time. One can also imagine combining the polymerases in a multistep reaction assay, using temperature as a parameter to control reaction sequence. As secondary structures in nucleic acids are highly sensitive to thermal destabilization, psychrophilic polymerases may be used for detection of such structures. Recent reports also suggest that dynamic modulation of liquid-frozen phase can also provide a new niche for psychrophilic polymerase applications (41, 42). The development of cold-adapted polymerases can greatly improve the capability of existing technologies and expand the temperature range of assays that one can perform.

In this study, we have also explored one of the long-standing questions in enzymology—is there a necessary trade-off between substrate specificity and activity? To address this question, we biochemically characterized bacterial DNA polymerase adapted to a wide range of environmental temperatures. Our results suggest that distinct temperature adaptation can differentially alter how the activity and fidelity of DNA polymerases change in response to environmental temperature fluctuations. We find that activity and fidelity have a log-linear relationship for many of the polymerases that we investigated. While the effect of temperature on Taq and T4 DNA polymerase error rate had been investigated (36, 43), we present the most comprehensive report of the temperature effect across polymerases on the single-molecule level, which enabled analysis of the temperature-dependent changes in the mutational spectrum. Our data show that psychrophilic and mesophilic polymerases tend to be more heat labile, whereas thermophilic polymerases, with the exception of Taq, are largely heat resistant, consistent with previous reports (44). Furthermore, increased reaction temperature substantially increased the error rate of PIPI and *Escherichia coli* DNA polymerase I (KleLFs) but not that of thermophilic polymerases such as Phusion and Q5. Nucleic acid analogs are of interest to a wide range of synthetic biology and biological engineering research as a means to expand orthogonal signals or building parts (45). This is often achieved through a combination of rational engineering and directed evolution on mesophilic or thermophilic polymerase templates (46, 47, 48, 49). It is tempting to speculate that using cold-adapted polymerases as a starting template may achieve higher substrate promiscuity and thus greater incorporation rate of unnatural nucleotides; however, further experiments are required to validate this conjecture. Engineering of psychrophilic enzymes may help improve most sequencing-by-synthesis sequencers available on the market as well as accelerate the application of nucleic acid analogs.

## Experimental procedures

### Polymerase cloning

*P. ingrahamii* 37 DNA Polymerases I (PIPI; IMG Gene ID: 639798289) sequence is provided by the Joint Genome Institute, Integrated Microbial Genomes & Microbiomes (IMG/G) portal (IMG Taxon ID: 639633052). The PIPI sequence was codon-optimized for *E. coli* expression, synthesized as a gene fragment (1845 bp), and then cloned into the pD454 T7 expression vector (ampicillin resistance) by ATUM (formerly DNA 2.0) to generate a tandem fusion construct with MBP separated by a single HRV 3C cleavage site. The cloning product was first subcloned in NEB-5α strains and then purified using ZymoPURE plasmid miniprep kits (Zymo Research). The sequence of the expression construct was verified with Sanger sequencing by Sequetech. The plasmid map is also provided in supplemental File S1.

### Polymerase purification

Several expression and purification conditions were attempted to optimize the yield and purity of PIPI. In the end, we generated MBP-PIPI (112.4 kDa) fusion gene on a pMAL-c5x vector and expressed it in ArcticExpress DE3 (Agilent Technologies), a bacterial expression cell line that expresses psychrophilic chaperones that promote proper protein folding during low temperature growth. Instead of LB, we grew the bacteria in a defined media M9ZB (supplemental File S2) at 37 ^°^C until *A*_600_ of 2.5 and induced expression with 1 M IPTG at 10 ^°^C or 16 ^°^C for 20 to 24 h. Cells were harvested in the lysis buffer (20 mM Tris HCl, pH 7.50, 10% glycerol, 50 mM NaCl, and 10 mM 2-mercaptoethanol) with the additions of 400 U of DNase I and 1X protease inhibitor cocktail (no EDTA; Thermo Fisher). Cells were lysed in a French press homogenizer by passing through at 500 psi once, and at 10,000 psi for three times. The clarified cell lysate was mixed with amylose resin at 4 ^°^C for 3 h. The resin was then washed with 20 column volumes of the chaperone removal buffer (20 mM Tris HCl, pH 7.50, 10% glycerol, 50 mM NaCl, 10 mM 2-mercaptoethanol, 50 mM KCl, 5 mM ATP) and washed with 10 column volumes of the lysis buffer (with no addition of DNase I or protease inhibitor). Bound proteins were eluted by incubating at 4 ^°^C for 3 h with 10 mM of maltose. MBP-PIPI was subjected to HRV-3C cleavage at 4 ^°^C overnight and was further purified in HiLoad 16/60 Superdex 200 preparatory-grade size-exclusion column (GE Healthcare) to separate fractions of polymerase proteins from cleaved MBP and protease. Purified proteins were eluted in the lysis buffer, concentrated in 30 kDa Amicon Ultra protein filters to ∼0.1 mg/ml, and then stored in −80 ^°^C until experiments.

### Protein quantification

Protein concentration was quantified by loading proteins on denaturing SDS-PAGE gel and stained with SYPRO Red following the manufacturer's recommended protocol (Thermo Fisher). Linear dynamic range of the quantification was established by separately staining a titration series of known amount of MBP-paramyosin-Sal. A secondary quantification measurement with Bioanalyzer Protein Analysis Kits (Agilent) provided similar concentration values as the SYPRO Red assay (data not shown).

### Polymerase activity assay

Polymerase activity from 5 ^°^C to 50 ^°^C was measured with the EvaEZ Fluorometric Polymerase Activity Assay Kit (Biotium) in the provided Tris buffer system based on the manufacturer's protocol. Polymerase was diluted to a concentration that is saturated by the substrate concentration. Briefly, the assay mix and polymerase were equilibrated to the reaction temperature separately for 5 min before mixing. Fluorescence signals of Eva dsDNA binding dye and ROX reference dye were measured in Bio-Rad 96-well qPCR machine for 1 h at a constant reaction temperature with 30-s interval. A positive control sample with saturating amount of KleExo- polymerase was included for each experiment to ensure that maximal fluorescence signals remained constant throughout measurement. The initial slope of the fluorescence gain for each polymerase was used to estimate the replication's steady-state speed.

### Gel-based primer extension assay

For measurement of subzero polymerase activity, we prepared 0.5 μl of polymerase prediluted to 5 nM in Mops buffer (pH 8.50). In parallel, we also prepared 9.5 μl of the primed extension mix containing a final concentration in 10-μl volume of the 10 nM primed template (TGATGGCGCCGTGACAGTGAAT) with 5′-ATTO 633 fluorescent label (/5ATTO633N/ATTCACTGTCACGGCGC; iDT) (supplemental File S1), 50 μM dNTP, 10 mM Mops, pH 8.50, 30% glycerol, 1.5 mM MgCl_2_, 0.1 mg/ml BSA, and 50 mM KCl. Reaction master mix and polymerase were separately equilibrated to −19 ^°^C on a TropiCooler (Boekel Scientific) for 30 min and then rapidly mixed using prechilled pipette tips. One microliter of the aliquot of the reaction was quenched in 9 μl of 90% formamide with 50 mM EDTA, pH 8.0, and heat-denatured at 95 ^°^C for 5 min before cooling on ice. Gels were imaged on Typhoon 9410 imager (GE).

### Molecular barcoding and library preparation

We purified the pUC19 plasmid template from a single, transformed clone of NEB-5α (New England Biolabs) grown in LB and 100 μg/ml ampicillin antibiotic. The plasmid's spontaneous mutagenesis rate is around ∼10^−7^ to 10^−8^ (50, 51), which is below the error rate of Q5 polymerase that we used for second-strand synthesis. About 1 μg of purified pUC19 was codigested with 10 units of SapI and NdeI at 37 ^°^C for 24 min to yield a 509-bp fragment. The digested fragment was purified twice with 0.35× (V:V) of AMPure XP beads to remove the backbone vector and restriction enzymes. Concentration and purity of the codigested pUC19 template were determined on Bioanalyzer using a high-sensitivity dsDNA quantification kit. Sequencing error in Illumina short-read sequencing occurs at a much higher frequency (∼10^−2^ to 10^−3^ per base) than polymerase replication error (10^−3^ to 10^−7^ per base). To confidently call out replication errors, sequencing errors have to be distinguished from replication errors.

We incorporated UMIs, a 15-bp region of random nucleotides, into each primer that serves as a molecular barcode (33) (supplemental File S1). This barcode is used to resolve the molecular origin of each sequenced read. As each molecule is sequenced multiple times, consensus sequences can be constructed to infer true replication errors. All reactions were conducted in a buffer made up of 10 mM Mops, pH 8.50, 30% glycerol, 1.5 mM MgCl_2_, 0.1 mg/ml BSA, and 50 mM KCl unless otherwise stated. We used the Mops buffer instead of the Tris-based buffer as it has a much smaller ΔpKa/ΔT scaling, which minimizes changes in buffer pH at different temperatures. The UMI index primer and pUC19 template were mixed at 100:1 M ratio, denatured at 95 ^°^C for 30 s, and annealed at 52 ^°^C for 2 min. We then preincubated the reaction mixture in a 96-well plate at the desired reaction temperature for 2 min before the addition of polymerase (the volume of the reaction mixture to the volume of polymerase is 19:1). The reaction time for each polymerase and condition was determined using fluorometric activity assays and qPCR measurement. The reaction was quenched with 50 mM EDTA and then purified with 1X AMPure XP beads. We then synthesized the complementary strand using the reverse UMI primer and Q5 DNA polymerase, a high-fidelity polymerase, in its native reaction buffer (NEB) by incubating at 72 ^°^C for 10 min, which is followed by EDTA quenching. We performed a second round of DNA purification with 1X AMPure XP beads and quantified molecular concentration of the resulting products using qPCR. A minimum of 20,000 barcoded molecules from each reaction was amplified in two rounds of 13 PCR cycles, with AMPure XP purification between each successive round of amplification (33). The Agilent Bioanalyzer dsDNA kit was used to assess the purity of the amplified product. Some reactions were repeated multiple times to obtain sufficient coverage for the analysis.

### Replication error analysis

The library was sequenced on MiSeq using the V3 2 × 300-bp chemistry kit to yield approximately 25 million reads. We sequenced some libraries multiple times to ensure sufficient sequencing coverage for the analysis. Each read is sorted into groups according to reaction indices. Each read is trimmed to a minimum of 150 length and Q score of 20 (Phred+33 quality score), ensuring that the composite reads will cover the entire template sequence. Reads with the same reaction indices are then grouped according to their UMI barcodes. Reads originating from the same replicated molecule would share the same UMI barcode. The consensus for each position was determined by 90% majority within the UMI family that shares the same base. Bases with less than 90% majority consensus may contain PCR-induced errors and thus are not included for downstream analysis. Consensus sequences with nonmatching bases in the first five positions are removed to avoid analysis of misprimed products. We observed that the apparent error rate for a polymerase decreased and plateaued as the consensus number increased, and we picked a minimum of five or ten consensus threshold for each molecule to improve accuracy in replication error calling. The consensus sequence from each UMI family is then aligned to the reference sequence using BWA-MEM (52). Substitution, insertion, and deletion errors are determined by comparing each UMI molecule's consensus sequence with the pUC19 reference sequence without counting ambiguous assignments. We did not observe any pre-existing mutation in the pUC19 reference that occurred across all reactions.

## Data availability

Processed data are available as Supplemental files (supplemental Files S3–S8). Raw and intermediate fastq files and supplemental files are deposited on Dryad: Xue, Yuan; Braslavsky, Ido; Quake, Stephen (2021), Temperature Effect on Polymerase Fidelity, Dryad, Dataset, https://doi.org/10.5061/dryad.76hdr7stv.

## Supporting information

This article contains supporting information.

## Conflict of interest

The authors declare that they have no conflicts of interest with the contents of this article.
