# Supplementary material for: Temperature effect on polymerase fidelity
Source: J Biol Chem. 2021 Oct 23;297(5):101270. doi: 10.1016/j.jbc.2021.101270 (PMC8592868; doi:10.1016/j.jbc.2021.101270)
Supplement: Figures S1–S3 [file mmc1.pdf]

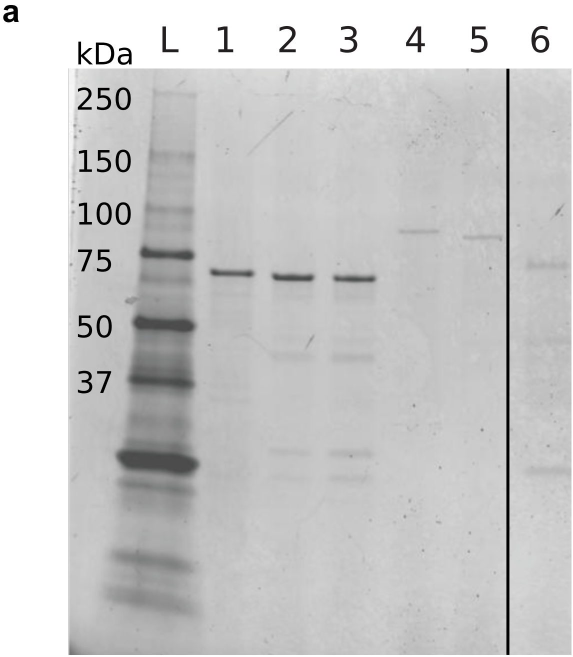

Supplemental figure 1. (a) SDS-PAGE analysis of purified DNA polymerases. Lane L: PrecisionPlus Ladder (Bio-rad). Lane 1: MBP-paramyosin protein control (NEB). Lane 2: Klenow Exo-(NEB). Lane 3: Klenow LF (NEB). Lane 4: Q5 (NEB). Lane 5: Taq (NEB). Lane 6: PIPI (69.9 kDa).

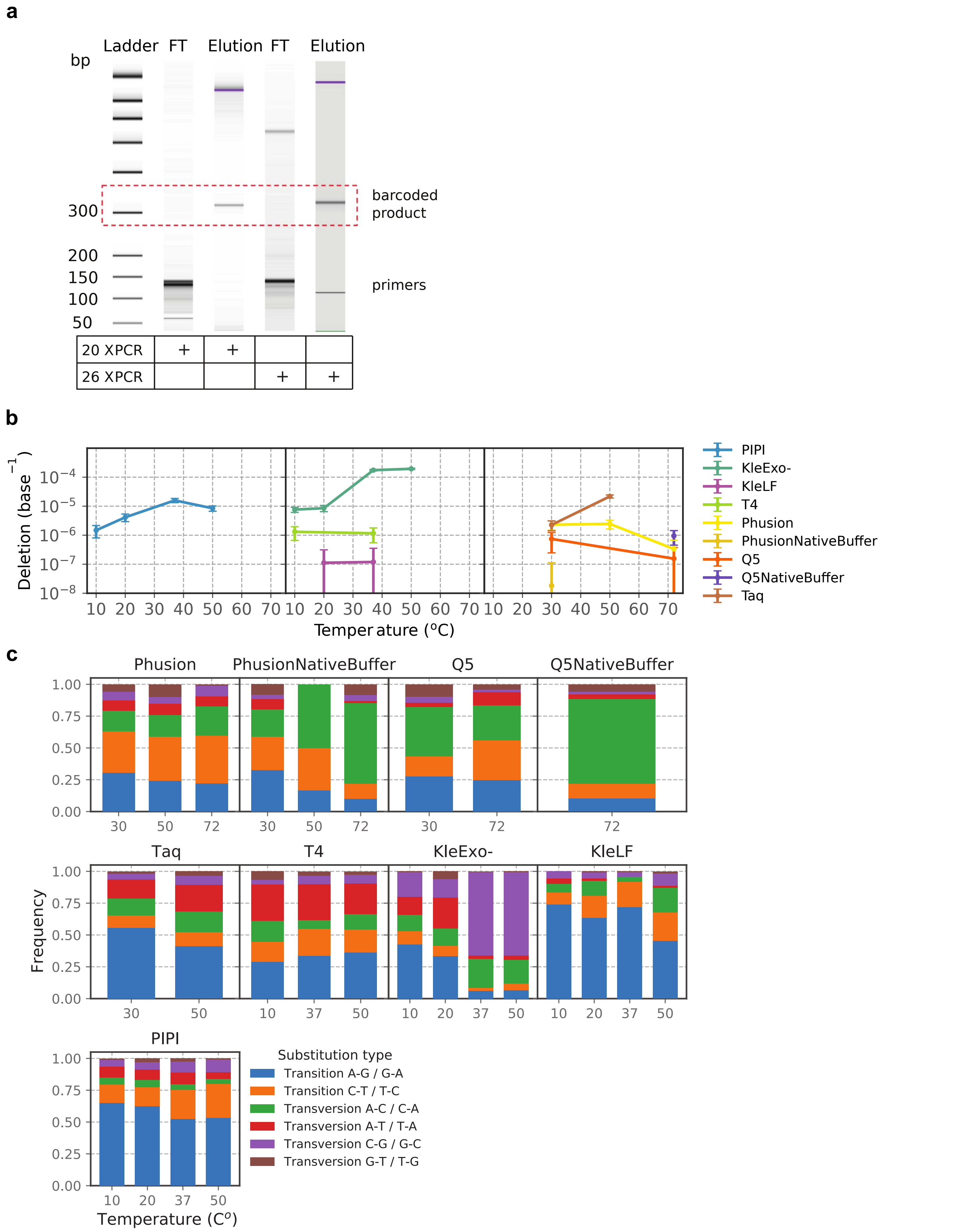

Supplemental figure 2. (a) High-sensitivity Bioanalyzer DNA (Agilent) assay of replicated products after 20 or 26 rounds of PCR amplification in the flow-through (FT) or elution fractions of Ampure purification output. **(b)** Deletion error rates (per base) of psychrophilic (left), mesophilic (center), and thermophilic (right) DNA polymerases as a function of reaction temperature. Data points with rates below the Q5 polymerase deletion rate baseline are excluded. **(c)** Substitution spectrum for all DNA polymerases tested in this study. The height of each colored bar reflects the frequency of a particular substitution type in all the substitution events observed for a polymerase reaction at the specified reaction temperature.

a

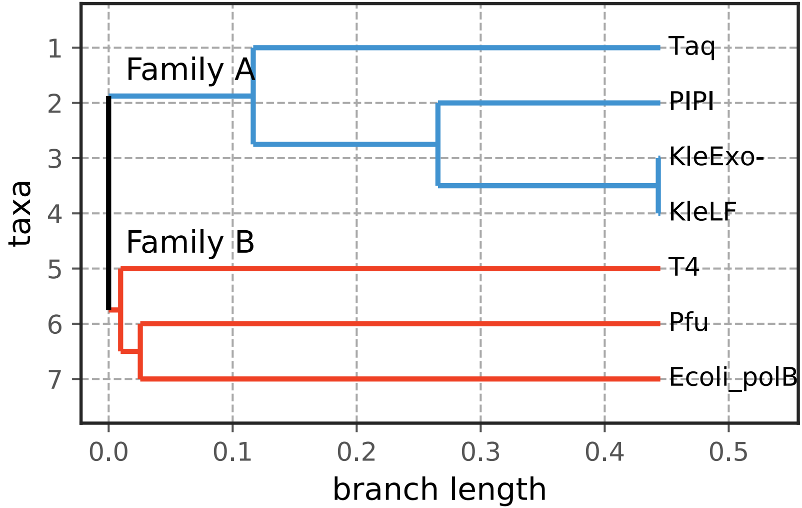

Supplemental figure 3. (a) Multiple sequence alignment (Clustal Omega) of peptide sequences for polymerases used in this study. While Phusion DNA polymerase sequence is undisclosed, it is expected to be a derivative of *Pyrococcus* DNA polymerase II (Pfu). Family A cluster consists of Taq, PIPI, and Klenow polymerases. Family B polymerases consist of T4, Pfu, and *E. coli* DNA polymerase II (Ecoli_polB).
