# Supplementary material for: Temperature effect on polymerase fidelity
Source: J Biol Chem. 2021 Oct 23;297(5):101270. doi: 10.1016/j.jbc.2021.101270 (PMC8592868; doi:10.1016/j.jbc.2021.101270)
Supplement: File S1 [file mmc2.pdf]

Supplemental text 1

pD454-PIPI (7096 bases, circular construct):

ctgggcggttctgataacgagtaatcgttaatccgcaaataacgtaaaaacccgcttcggcgggtttttttatgggggggagtttagggaaagagcatttgtcagaatatttaagggcgcc
tgtcactttgcttgatatatgagaattatttaaccttataaatgagaaaaaagcaacgcactttaaataagatacgttgctttttcgattgatgaacacctataattaaactattcatctattattta
tgatttttttgtatatacaatatttctagtttgttaaagagaattaagaaaataaatctcgaaaataataaagggaaaatcagtttttgatatcaaaattatacatgtcaacgataatacaaaatat
aatacaaactataagatgttatcagtatttattatcatttagaataaattttgtgtcgcccttccgcgaaattaatacgactcactataggggaattgtgagcggataacaattcccctctaga
aataattttgtttaactttttgagaccttaaagaaggagatataaaATGCATCATCACCACCATCATAAAACTGAAGAAGGTAAACTGGTAAT
CTGGATTAACGGCGATAAAGGCTATAACGGTTTGGCTGAAGTCGGTAAGAAATTCGAGAAAGATACCGGAATTA
AAGTCACCGTTGAGCATCCGGATAAACTGGAAGAGAAATTCCCACAGGTTGCGGCAACTGGCGATGGCCCTGA
CATTATCTTCTGGGCACACGACCGCTTTGGTGGCTACGCTCAATCTGGCCTGTTGGCTGAAATCACCCCGGACA
AAGCGTTCCAGGACAAGCTGTATCCGTTTACCTGGGATGCCGTACGTTACAACGGCAAGCTGATTGCTTACCCG
ATCGCTGTTGAAGCGTTATCGCTGATTTATAACAAAGATCTGCTGCCGAACCCGCCAAAAACCTGGGAAGAGAT
CCCGGCGCTGGATAAAGAACTGAAAGCGAAAGGTAAGAGCGCGCTGATGTTCAACCTGCAAGAACCGTACTT
CACCTGGCCGCTGATTGCTGCTGACGGGGGTTATGCGTTCAAGTATGAAAACGGCAAGTACGACATTAAAGAC
GTGGGCGTGGATAACGCTGGCGCGAAAGCGGGTCTGACCTTCCTGGTTGACCTGATTAAAAACAAACACATGA
ATGCAGACACCGATTACTCCATCGCAGAAGCTGCCTTTAATAAAGGCGAAACAGCGATGACCATCAACGGCCC
GTGGGCATGGTCCAACATCGACACCAGCAAAGTGAATTATGGTGTAACGGTACTGCCGACCTTCAAGGGTCAA
CCATCCAAACCGTTCGTTGGCGTGCTGAGCGCAGGTATTAACGCCGCCAGTCCGAACAAAGAGCTGGCAAAAG
AGTTCCTCGAAAACTATCTGCTGACTGATGAAGGTCTGGAAGCGGTTAATAAAGACAAACCGCTGGGTGCCGT
AGCGCTGAAGTCTTACGAGGAAGAGTTGGCGAAAGATCCACGTATTGCCGCCACTATGGAAAACGCCCAGAA
AGGTGAAATCATGCCGAACATCCCGCAGATGTCCGCTTTCTGGTATGCCGTGCGTACTGCGGTGATCAACGCCG
CCAGCGGTCGTCAGACTGTCGATGAAGCCCTGAAAGACGCGCAGACTAATCTGGAAGTTCTTTTTAATTCGAG
CTCGAACAACAACAACAATAACAATAACAACAACCTCGGGATCGAGGGAAGGATTTCACATATGCTGGAAGTT
CTTTTTCAGGGTCCT**ATGATCGATCGTAGCGGCTACAAAACCATCTATACGCAAGGCCAACTGACCCAGTG**
GATCGCGAAGTTAGAGGCCGCTGAGCTGTTTAGCTTCGATACCGAAACCACCTCGTTGGACTACATGCA
AGCGCGTATCGTGGGCTTGAGCTTTGCGGTTCAGAGCAATGTTGGTAGCGATGAGCAGCCGATTATCGA
AGCCGCCTATCTGCCACTGGCCCACGACTACATCGATGCACCGAAACAGCTCGATCTGACGACCACTCT
GGAGAAACTGCGCCCGCTGCTGGAGTCTGAGAAATATAAAAAAGTCGGTCAGAACCTGAAATATGACCG
TAGCGTCTTGCTGAATCACGGTATCGAGCTGAAGGGTATTAAGTTCGACACGATGCTGGAGAGCTATGT
GCTGGATTCCACCGGTCGTCATGACATGGACACCCTGGCGTTAAAGTACCTGGGCCATCAGTGCATCAG
CTTCGAAGAAATTGCTGGTAAGGGTAAAAAGCAACTGCCGTTCAATCAGATCTCCATCATTGAAGCGGC
ACCGTACGCGGCGGAAGATGCAGATGTTACGTTGCGTCTGCACTTGCAGTTGTTTGAGCAACTGAGCGC
TGAGCCGAAACTTCTGAGCGTGTTTAACAATATCGAGCTGCCGCTGTTGACGGTCCTGTCCGATATTGA
GCGTGGTGGCGTTGAGATTGATTCGGACCTGTTGACGAAACAAAGCGCGGAAATTGGCAAGCGTCTGC
TGGAACTGGAAGCACTGGCTTATGAAGAGGCCGGCAAACCGTTCAACCTGAGCTCTCCGAAACAGCTG
CAGACCATTCTGTACGATGAGCTGGAACTGCCGGTTCTGAAGAAAACCCCGAAGGGTGCGCCGAGCAC
CGCCGAGGAAGTGCTGCAAGAACTGGCATTGACGTACCCTCTGCCAAAGCTGATCATCGAGCACCGTG
GTCTG12AGCAAACTGAAAAGCACCTATACGGACAAGCTGCCGAAAATGGTTAATGAAAAGACTGGCCG
TCTGCACACCAGCTATCAACAAGCCGTGACGGTGACGGGTCGCCTGTCCTCTACGGACCCGAATCTGCA
GAATATCCCGATTCGCTCTGCAGAGGGCCGTCGTATCCGTCAAGCGTTCATTGCGCAGCCTGGTTACAA
GATCGTCGCGGCGGACTACAGCCAAATCGAGTTGCGTATCATGGCGCATCTGAGCCAGGACAAGGGCC
TGCTGGACGCATTCTCCACCGGCAAAGACATTCACAAAGCAACCGCGAGCGAAGTTTTCAGCGTTCCGC
TGGATGAAGTCACTACCGAGCAGCGCCGTTCCGCGAAGGCTATCAACTTTGGCTTGATCTATGGCATGA
GCGCGTTCGGCCTGGCGAAGCAACTCAACATTGGTCGCCACGAAGCACAGCTGTATATGGACAAATATT
TTAACCGTTACCCGGGTGTGCTGATTTACATGGAAGATACCCGCAGCCTGGCGAACGAAAAGGCTTACG
TTGAAACCATTCTGGGTAGACGTTTGCAGCTGCCGAATATTAAGAGCCGCAATGGTATGCTGAAAAAAG
CCGCGGAGCGTGCCGCGATTAACGCGCCGATGCAAGGTACCGCTGCGGATATTATCAAAAAAGCAATGA
TTGACATGGCAGACTGGATTGCGCAGAAGTCCCCGGGTAGCGTTCAAATGCTGATGCAAGTGCATGATG
AACTGGTCTTTAGCATTAAGGAAGAATTGGTCGAGAGCTACACGAAAGAAATTCAGGCCATCATGGCAA
AAGCCGCAGATCTGGATGTGCCACTGATCGCTGACGCCGGTGTCGGTGATAACTGGGACGAAGCGCAC
TAAggttagataatagggtctcacccccctagcataaccccttggggcctctaaacgggtcttgaggggttttttgcccctgagacgcgtcaatcgagttcgtacctaagggcgacac
cccctaattagcccgggcgaaaggcccagtctttcgactgagcctttcgttttatttgatgcctggcagttccctactctcgcatggggagtccccacactaccatcggcgctacggcgt
ttcacttctgagttcggcatggggtcaggtgggaccaccgcgctactgccgccaggcaaacaaggggtgttatgagccatattcaggtataaatgggctcgcgataatgttcagaatt
ggttaattggttgtaacactgacccctatttgtttatttttctaaatacattcaaatatgtatccgctcatgagacaataaccctgataaatgcttcaataatattgaaaaaggaagaatatgagt
attcaacatttccgtgtcgcccttattcccttttttgcggcattttgccttcctgtttttgctcacccagaaacgctggtgaaagtaaaagatgctgaagatcagttgggtgcacgagtgggtt
acatcgaactggatctcaacagcggtaagatccttgagagttttcgccccgaagaacgttttccaatgatgagcacttttaaagttctgctatgtggcgcggtattatcccgtattgacgc
cgggcaagagcaactcggtcgccgcatacactattctcagaatgacttggttgagtactcaccagtcacagaaaagcatcttacggatggcatgacagtaagagaattatgcagtgct

gccataaccatgagtgataacactgcggccaacttacttctgacaacgatcggaggaccgaaggagctaaccgcttttttgcacaacatgggggatcatgtaactcgccttgatcgttg
ggaaccggagctgaatgaagccataccaaacgacgagcgtgacaccacgatgcctgtagcgatggcaacaacgttgcgcaaactattaactggcgaactacttactctagcttccc
ggcaacaattaatagactggatggaggcggataaagttgcaggaccacttctgcgctcggcccttccggctggctggtttattgctgataaatccggagccggtgagcgtggttctcg
cggtatcatcgcagcgctggggccagatggtaagccctcccgtatcgtagttatctacacgacggggagtcaggcaactatggatgaacgaaatagacagatcgctgagataggtg
cctcactgattaagcattggtaagcggcgcgccatcgaatggcgcaaaacctttcgcggtatggcatgatagcgcccggaagagagtcaattcagggtggtgaatatgaaaccagt
aacgttatacgatgtcgcagagtatgccggtgtctcttatcagaccgtttcccgcgtggtgaaccaggccagccacgtttctgcgaaaacgcgggaaaaagtggaagcggcgatgg
cggagctgaattacattcccaaccgcgtggcacaacaactggcgggcaaacagtcgttgctgattggcgttgccacctccagtctggccctgcacgcgccgtcgcaaattgtcgcg
gcgattaaatctcgcgccgatcaactgggtgccagcgtggtggtgtcgatggtagaacgaagcggcgtcgaagcctgtaaagcggcggtgcacaatcttctcgcgcaacgcgtca
gtgggctgatcattaactatccgctggatgaccaggatgccattgctgtggaagctgcctgcactaatgttccggcgttatttcttgatgtctctgaccagacacccatcaacagtattatt
ttctcccatgaggacggtacgcgactgggcgtggagcatctggtcgcattgggtcaccagcaaatcgcgctgttagcgggcccattaagttctgtctcggcgcgtctgcgtctggctg
gctggcataaatatctcactcgcaatcaaattcagccgatagcggaacgggaaggcgactggagtgccatgtccggttttcaacaaaccatgcaaatgctgaatgagggcatcgttc
ccactgcgatgctggttgccaacgatcagatggcgctgggcgcaatgcgcgccattaccgagtccgggctgcgcgttggtgcggatatctcggtagtgggatacgacgataccga
agatagctcatgttatatcccgccgttaaccaccatcaaacaggattttcgcctgctggggcaaaccagcgtggaccgcttgctgcaactctctcagggccaggcggtgaagggcaa
tcagctgttgccagtctcactggtgaaaagaaaaaccaccctggcgcccaatacgcaaaccgcctctccccgcgcgttggccgattcattaatgcagctggcacgacaggtttcccg
actggaaagcgggcagtgactcatgaccaaaatcccttaacgtgagttacgcgcgcgtcgttccactgagcgtcagaccccgtagaaaagatcaaaggatcttcttgagatcctttttt
tctgcgcgtaatctgctgcttgcaaacaaaaaaaccaccgctaccagcggtggtttgtttgccggatcaagagctaccaactctttttccgaaggtaactggcttcagcagagcgcaga
taccaaatactgttcttctagtgtagccgtagttagcccaccacttcaagaactctgtagcaccgcctacatacctcgctctgctaatcctgttaccagtggctgctgccagtggcgataa
gtcgtgtcttaccgggttggactcaagacgatagttaccggataaggcgcagcggtcgggctgaacggggggttcgtgcacacagcccagcttggagcgaacgacctacaccga
actgagatacctacagcgtgagctatgagaaagcgccacgcttcccgaagggagaaaggcggacaggtatccggtaagcggcagggtcggaacaggagagcgcacgaggga
gcttccagggggaaacgcctggtatctttatagtcctgtcgggtttcgccacctctgacttgagcgtcgatttttgtgatgctcgtcaggggggcggagcctatggaaaaacgccagca
acgcggcctttttacggttcctggccttttgctggccttttgctcacatgttctttcctgcgttatcccctgattctgtggataaccgtattaccgcctttgagtgagctgataccgctcgccgc
agccgaacgaccgagcgcagcgagtcagtgagcgaggaagcggaaggcgagagtagggaactgccaggcatcaaactaagcagaaggcccctgacggatggcctttttgcgt
ttctacaaactctttctgtgttgtaaaacgacggccagtcttaagctcgggcccc

*upper cases: open reading frame of expression construct.
*upper cases + **boldened**: coding sequence for PIPI.

pUC19 (2686 bases, circular construct):
gacgaaagggcctcgtgatacgcctatttttataggttaatgtcatgataataatggtttcttagacgtcaggtggcacttttcggggaaatgtgcgcggaacccctatttgtttatttttcta
aatacattcaaatatgtatccgctcatgagacaataaccctgataaatgcttcaataatattgaaaaaggaagagtatgagtattcaacatttccgtgtcgcccttattcccttttttgcggca
ttttgccttcctgtttttgctcacccagaaacgctggtgaaagtaaaagatgctgaagatcagttgggtgcacgagtgggttacatcgaactggatctcaacagcggtaagatccttgag
agttttcgccccgaagaacgttttccaatgatgagcacttttaaagttctgctatgtggcgcggtattatcccgtattgacgccgggcaagagcaactcggtcgccgcatacactattctc
agaatgacttggttgagtactcaccagtcacagaaaagcatcttacggatggcatgacagtaagagaattatgcagtgctgccataaccatgagtgataacactgcggccaacttactt
ctgacaacgatcggaggaccgaaggagctaaccgcttttttgcacaacatgggggatcatgtaactcgccttgatcgttgggaaccggagctgaatgaagccataccaaacgacga
gcgtgacaccacgatgcctgtagcaatggcaacaacgttgcgcaaactattaactggcgaactacttactctagcttcccggcaacaattaatagactggatggaggcggataaagtt
gcaggaccacttctgcgctcggcccttccggctggctggtttattgctgataaatctggagccggtgagcgtgggtctcgcggtatcattgcagcactggggccagatggtaagccct
cccgtatcgtagttatctacacgacggggagtcaggcaactatggatgaacgaaatagacagatcgctgagataggtgcctcactgattaagcattggtaactgtcagaccaagttta
ctcatatatactttagattgatttaaaacttcatttttaatttaaaaggatctaggtgaagatcctttttgataatctcatgaccaaaatcccttaacgtgagttttcgttccactgagcgtcagac
cccgtagaaaagatcaaaggatcttcttgagatcctttttttctgcgcgtaatctgctgcttgcaaacaaaaaaaccaccgctaccagcggtggtttgtttgccggatcaagagctaccaa
ctctttttccgaaggtaactggcttcagcagagcgcagataccaaatactgttcttctagtgtagccgtagttaggccaccacttcaagaactctgtagcaccgcctacatacctcgctct
gctaatcctgttaccagtggctgctgccagtggcgataagtcgtgtcttaccgggttggactcaagacgatagttaccggataaggcgcagcggtcgggctgaacggggggttcgtg
cacacagcccagcttggagcgaacgacctacaccgaactgagatacctacagcgtgagctatgagaaagcgccacgcttcccgaagggagaaaggcggacaggtatccggtaa
gcggcagggtcggaacaggagagcgcacgagggagcttccagggggaaacgcctggtatctttatagtcctgtcgggtttcgccacctctgacttgagcgtcgatttttgtgatgctc
gtcaggggggcggagcctatggaaaaacgccagcaacgcggcctttttacggttcctggccttttgctggccttttgctcacatgttctttcctgcgttatcccctgattctgtggataacc
gtattaccgcctttgagtgagctgataccgctcgccgcagccgaacgaccgagcgcagcgagtcagtgagcgaggaagcggaagagcgcccaatacgcaaaccgcctctcccc
gcgcgttggccgattcattaatgcagctggcacgacaggtttcccgactggaaagcgggcagtga**GCGCAACGCAATTAATGTGAGTTAGCTCAC
TCATTAGGCACCCCAGGCTTTACACTTTATGCTTCCGGCTCGTATGTTGTGTGGAATTGTGAGCGGATAA
CAATTTCACACAGGAAACAGCTATGACCATGATTACGCCAAGCTTGCATGCCTGCAGGTCGACTCTAGA
GGATCCCCGGGTACCGAGCTCGAATTCACTGGCCGTCGTTTTACAACGTCGTGACTGGGAAAACCCTGG
CGTTACCCAACTTAATCGCCTTGCAGCACATCCCCCTTTCGC**cagctggcgtaatagcgaagaggcccgcaccgatcgcccttcccaa
cagttgcgcagcctgaatggcgaatggcgcctgatgcggtattttctccttacgcatctgtgcggtatttcacaccgcatatggtgcactctcagtacaatctgctctgatgccgcatagt
taagccagccccgacacccgccaacacccgctgacgcgccctgacgggcttgtctgctcccggcatccgcttacagacaagctgtgaccgtctccgggagctgcatgtgtcagag
gttttcaccgtcatcaccgaaacgcgcga

*upper cases + **boldened**: reference template for polymerase error rate measurement.

atto633_RP (17 bases):
/5ATTO633N/**ATTCACTGTCACGGCGC**

extension_template (22 bases):
TGATG**GCGCCGTGACAGTGAAT**

Unique molecule indexed (UMI) primers for single-molecule polymerase error measurement:

Name	Sequence	Purification	Length
**YX_F00**	ACACTCTTTCCCTACACGACGCTCTTCCGATCT**CTGT**NNNNNNNNNNNNNNNgcgcaacgcaattaatgtga	PAGE	72
**YX_F01**	ACACTCTTTCCCTACACGACGCTCTTCCGATCT**ACGTA**NNNNNNNNNNNNNNNgcgcaacgcaattaatgtga	PAGE	73
**YX_F02**	ACACTCTTTCCCTACACGACGCTCTTCCGATCT**ATTGAT**NNNNNNNNNNNNNNNgcgcaacgcaattaatgtga	PAGE	74
**YX_F03**	ACACTCTTTCCCTACACGACGCTCTTCCGATCT**AGACTGG**NNNNNNNNNNNNNNNgcgcaacgcaattaatgtga	PAGE	75
**YX_F04**	ACACTCTTTCCCTACACGACGCTCTTCCGATCT**CAGTCATC**NNNNNNNNNNNNNNNgcgcaacgcaattaatgtga	PAGE	76
**YX_F05**	ACACTCTTTCCCTACACGACGCTCTTCCGATCT**CGTCCACTG**NNNNNNNNNNNNNNNgcgcaacgcaattaatgtga	PAGE	77
**YX_F06**	ACACTCTTTCCCTACACGACGCTCTTCCGATCT**TACTGCATAC**NNNNNNNNNNNNNNNgcgcaacgcaattaatgtga	PAGE	78
**YX_F07**	ACACTCTTTCCCTACACGACGCTCTTCCGATCT**TCTACTGAGTA**NNNNNNNNNNNNNNNgcgcaacgcaattaatgtga	PAGE	79
**YX_F08**	ACACTCTTTCCCTACACGACGCTCTTCCGATCT**TGCAGCTCTAGT**NNNNNNNNNNNNNNNgcgcaacgcaattaatgtga	PAGE	80
**YX_F09**	ACACTCTTTCCCTACACGACGCTCTTCCGATCT**GTCCGGCAATCGG**NNNNNNNNNNNNNNNgcgcaacgcaattaatgtga	PAGE	81
**YX_F10**	ACACTCTTTCCCTACACGACGCTCTTCCGATCT**GAAGTCAGCGTACG**NNNNNNNNNNNNNNNgcgcaacgcaattaatgtga	PAGE	82
**YX_R00**	CGTGATGTGACTGGAGTTCAGACGTGTGCTCTTCCGATCT**GGTA**NNNNNNNNNNNNNNNgcgaaagggggatgtgc	PAGE	76
**YX_R01**	CGTGATGTGACTGGAGTTCAGACGTGTGCTCTTCCGATCT**TCAGT**NNNNNNNNNNNNNNNgcgaaagggggatgtgc	PAGE	77
**YX_R02**	CGTGATGTGACTGGAGTTCAGACGTGTGCTCTTCCGATCT**TGCCTG**NNNNNNNNNNNNNNNgcgaaagggggatgtgc	PAGE	78
**YX_R03**	CGTGATGTGACTGGAGTTCAGACGTGTGCTCTTCCGATCT**TAGAGCA**NNNNNNNNNNNNNNNgcgaaagggggatgtgc	PAGE	79
**YX_R04**	CGTGATGTGACTGGAGTTCAGACGTGTGCTCTTCCGATCT**CTAGCTGC**NNNNNNNNNNNNNNNgcgaaagggggatgtgc	PAGE	80
**YX_R05**	CGTGATGTGACTGGAGTTCAGACGTGTGCTCTTCCGATCT**CATCCTCGA**NNNNNNNNNNNNNNNgcgaaagggggatgtgc	PAGE	81
**YX_R06**	CGTGATGTGACTGGAGTTCAGACGTGTGCTCTTCCGATCT**GTCGACTGTC**NNNNNNNNNNNNNNNgcgaaagggggatgtgc	PAGE	82
**YX_R07**	CGTGATGTGACTGGAGTTCAGACGTGTGCTCTTCCGATCT**GCGTCGATAGT**NNNNNNNNNNNNNNNgcgaaagggggatgtgc	PAGE	83
**YX_R08**	CGTGATGTGACTGGAGTTCAGACGTGTGCTCTTCCGATCT**ATGCGACACTCA**NNNNNNNNNNNNNNNgcgaaagggggatgtgc	PAGE	84
**YX_R09**	CGTGATGTGACTGGAGTTCAGACGTGTGCTCTTCCGATCT**AGTACAGCTTAGA**NNNNNNNNNNNNNNNgcgaaagggggatgtgc	PAGE	85
**YX_R10**	CGTGATGTGACTGGAGTTCAGACGTGTGCTCTTCCGATCT**ACTAACTAGAGTCG**NNNNNNNNNNNNNNNgcgaaagggggatgtgc	PAGE	86
